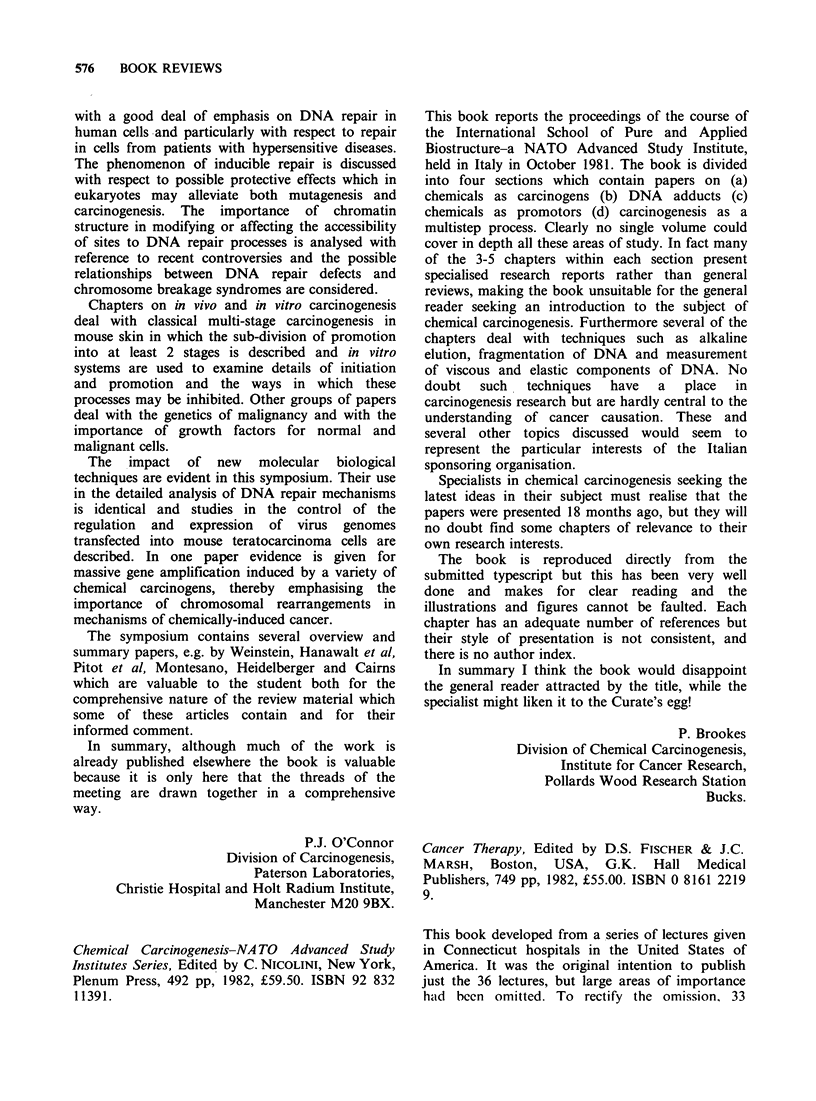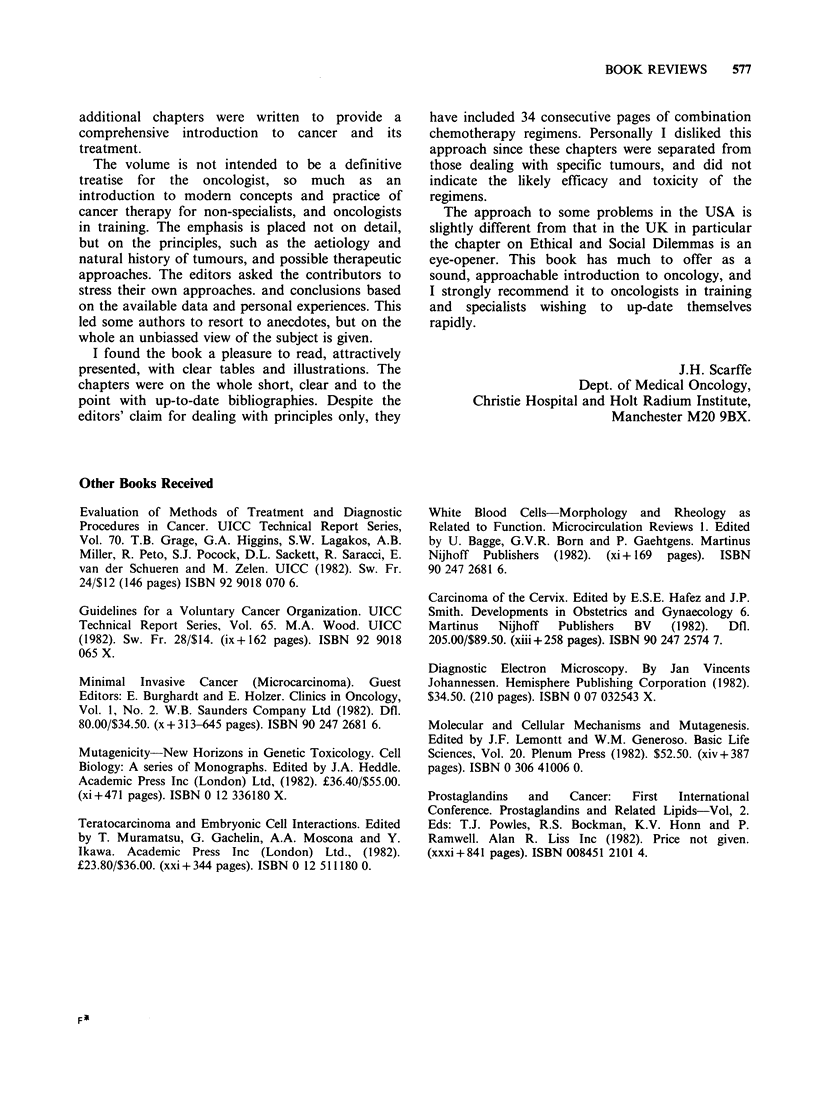# Cancer Therapy

**Published:** 1983-04

**Authors:** J.H. Scarffe


Christie Hospital and Holt Radium Institute,

Manchester M20 9BX.

*Cancer Therapy*, Edited by D.S. Fischer & J.C. Marsh, Boston, USA, G.K. Hall Medical Publishers, 749 pp, 1982, £55.00. ISBN 0 8161 2219 9.

This book developed from a series of lectures given in Connecticut hospitals in the United States of America. It was the original intention to publish just the 36 lectures, but large areas of importance had been omitted. To rectify the omission, 33

additional chapters were written to provide a comprehensive introduction to cancer and its treatment.

The volume is not intended to be a definitive treatise for the oncologist, so much as an introduction to modern concepts and practice of cancer therapy for non-specialists, and oncologists in training. The emphasis is placed not on detail, but on the principles, such as the aetiology and natural history of tumours, and possible therapeutic approaches. The editors asked the contributors to stress their own approaches. and conclusions based on the available data and personal experiences. This led some authors to resort to anecdotes, but on the whole an unbiassed view of the subject is given.

I found the book a pleasure to read, attractively presented, with clear tables and illustrations. The chapters were on the whole short, clear and to the point with up-to-date bibliographies. Despite the editors' claim for dealing with principles only, they have included 34 consecutive pages of combination chemotherapy regimens. Personally I disliked this approach since these chapters were separated from those dealing with specific tumours, and did not indicate the likely efficacy and toxicity of the regimens.

The approach to some problems in the USA is slightly different from that in the UK in particular the chapter on Ethical and Social Dilemmas is an eye-opener. This book has much to offer as a sound, approachable introduction to oncology, and I strongly recommend it to oncologists in training and specialists wishing to up-date themselves rapidly.

J.H. Scarffe
Dept. of Medical Oncology,
Christie Hospital and Holt Radium Institute,
Manchester M20 9BX.